# The emerging role of human cytomegalovirus infection in human carcinogenesis: a review of current evidence and potential therapeutic implications

**DOI:** 10.18632/oncotarget.27016

**Published:** 2019-07-02

**Authors:** Cecilia Söderberg Nauclér, Jürgen Geisler, Katja Vetvik

**Affiliations:** ^1^ Department of Medicine, Unit of Microbial Pathogenesis, Center for Molecular Medicine, Karolinska Institutet, Solna, Stockholm, Sweden; ^2^ Department of Oncology, Akershus University Hospital (AHUS), Lørenskog, Norway; ^3^ Institute of Clinical Medicine, University of Oslo, Oslo, Norway; ^4^ Department of Breast and Endocrine Surgery, AHUS, Lørenskog, Norway

**Keywords:** human cytomegalovirus, HCMV, oncovirus, cancer, glioblastoma

## Abstract

It is well-established that infections with viruses harboring oncogenic potential increase the cancer risk. Virus induced oncogenic processes are influenced by a complex and unique combination of host and environmental risk factors that are currently not fully understood. Many of the oncogenic viruses exhibit a prolonged, asymptomatic latency after a primary infection, and cause cancer in only a minority of carriers. From an epidemiologic point of view, it is therefore difficult to determine their role in cancer development. However, recent evidence suggests a neoplastic potential of one additional ubiquitous virus; human Cytomegalovirus (HCMV). Emerging data presents HCMV as a plausible cancer-causing virus by demonstrating its presence in >90% of common tumor types, while being absent in normal tissue surrounding the tumor. HCMV targets many cell types in tumor tissues, and can cause all the ten proposed hallmarks of cancer. This virus exhibits cellular tumor-promoting and immune-evasive strategies, hijacks proangiogenic and anti-apoptotic mechanisms and induces immunosuppressive effects in the tumor micro-environment. Recognizing new cancer-causing mechanisms may increase the therapeutic potential and prophylactic options for virus associated cancer forms. Such approaches could limit viral spread, and promote anti-viral and immune controlling strategies if given as add on to standard therapy to potentially improve the prognosis of cancer patients. This review will focus on HCMV-related onco-viral mechanisms and the potential of HCMV as a new therapeutic target in HCMV positive cancer forms.

## INTRODUCTION

**Figure 1:**
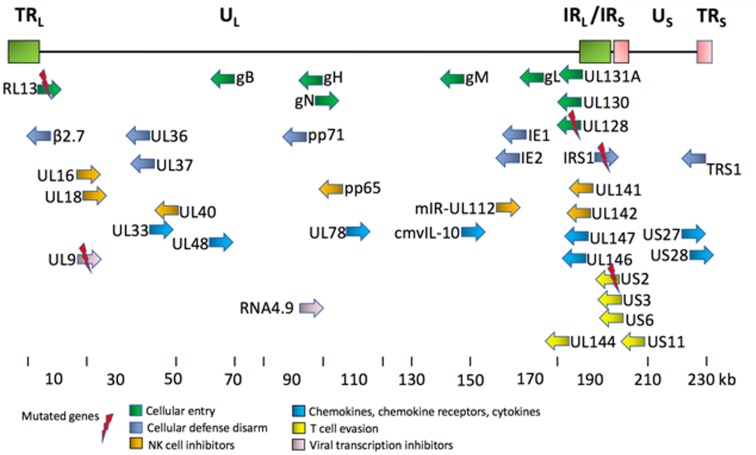
Key proteins encoded by HCMV genome [8, 119]. This simplified diagram shows the HCMV genome, and its key gene products, their relative position and orientation, and their functional classifications [8]. The common places for mutations in the clinical strains are RL13 gene, (DB, Toledo, TB40/E, Merlin, Davis), UL9 gene (DB, Toledo), UL128 gene (Toledo, TB40/E), IRS gene (TB40/E), and US2 (TB40/E). The gene names in HCMV genome are not always placed according to their location due to historical precedence in nomenclature assignments and rearrangements among the strains. The HCMV genome contains from the left TRL1-14 (green box), UL1-147, IRL 14-1 (green box), IRS1 (red box), US1-36, and TRS1 (red box).

**Figure 2:**
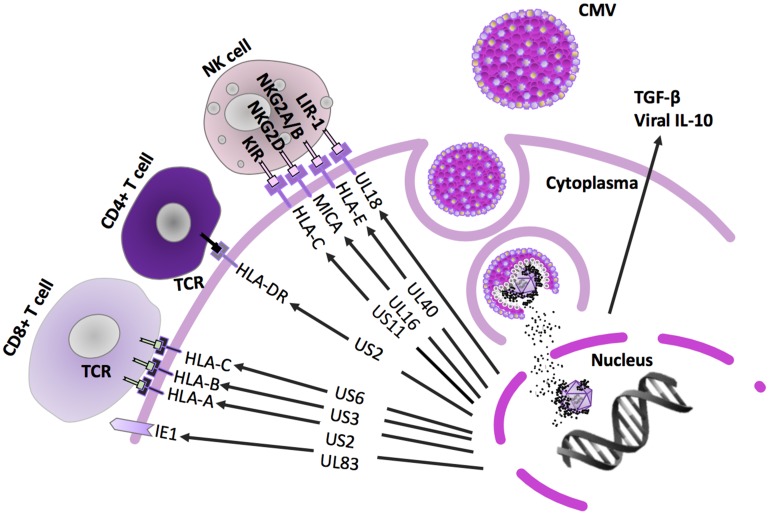
Innate and adaptive immune pathways inhibited by HCMV. After entrance in the target cells, HCMV encoded proteins downregulate intrinsic, but also innate and adaptive immune pathways to avoid elimination by the immune system. Viral lytic glycoproteins US2-US11 downregulate HLA class I- and class II-dependent antigen presentation to T cells [49, 50]. This affects both CD8+ cytotoxic tumor elimination and activation of CD4+ T-cell responses including activation of humoral immune response and B cells. In parallel, to counteract the NK cell dependent cell lysis, the HCMV encoded HLA class I homolog UL18 can bind to the NK cell inhibitory receptor NKG2A/CD94 and expression of HLA-E, a non-classical HLA protein, is upregulated [51–53]. HCMV enhances production of the immunosuppressive factors, such as T reg cells expressed membrane-bound transforming growth factor (TGF)-β and IL-10, where also TGF-β directly contributes to inhibit NK cell effector functions. In addition, HCMV exhibits a powerful immunosuppressive effect by expressing a cmvIL-10 (*UL111A* gene), which can promote maturation of pro-tumoral M2 macrophages and counteract the proper maturation of dendritic cells [56, 57]. Natural Killer (NK)-cells are able to eliminate virus-infected and altered cells, and they produce a number of important cytokines that stimulate the antiviral and antitumor adaptive immune response, especially interferon gamma [120]. Epigenetic reprogramming and disarmament of NK-cells is well-established HCMV mediated effect and may be a critical contributor during the carcinogenic process [121].

**Figure 3:**
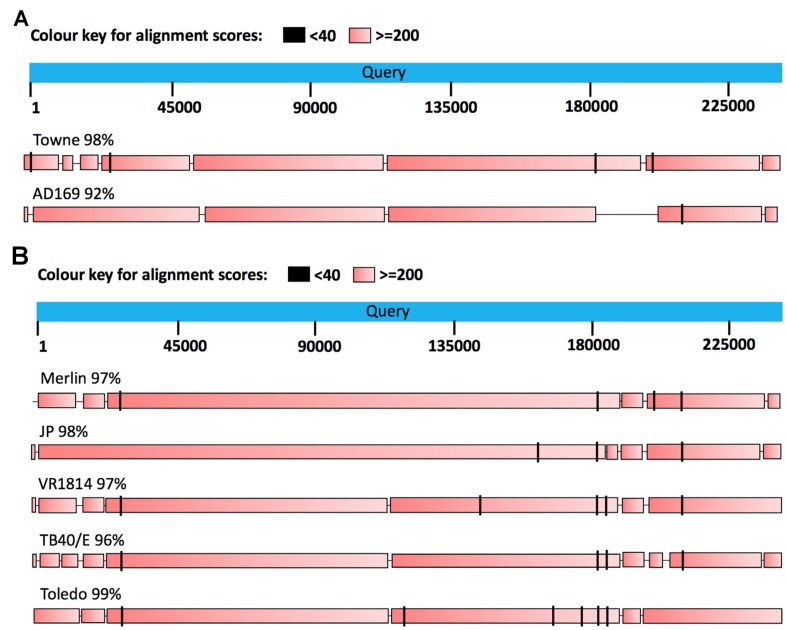
(**A**) BLAST alignment of two human cytomegalovirus laboratory strains, Towne and AD169, with the DB clinical strain [8]. The laboratory strains have been extensively passaged in fibroblasts as vaccine candidates. They show 98% and 92% similarity, respectively, with the DB clinical strain. The Towne laboratory strain consists of a block of ORFs (UL147-151), that is not present in AD169. (**B**) BLAST alignment to compare the DB clinical strain with five other strains. These strains are considered as clinical isolates, since they have been passaged to a limited extent in the laboratory (Merlin, JP, VR1814, TB40/E and Toledo). The DB sequence is reported to be 99% similar to Toledo sequence, and 96–98% similar to other clinical isolates. Toledo, was isolated from the urine of a congenitally HCMV infected child [122]; DB was isolated from a pregnant woman in France [123]; TB40/E was isolated from a throat wash of a bone marrow transplant patient [124]; JP was isolated from prostate tissue of post mortem AIDS patient [123]; Merlin was isolated from urine of congenitally infected infant [125]; and VR1814 was isolated from cervical secretion of a pregnant woman with a primary HCMV infection [126] Each of the clinical isolates can replicate in several cell types in addition to fibroblasts, whereas the replication of the laboratory strains is limited to fibroblasts.

Human cytomegalovirus (HCMV) is an opportunistic DNA virus that infects a majority of the adult population worldwide [1], and is by far the largest and most complex of all human herpesviruses [2]. HCMV is transmitted by all body fluids including saliva and breast milk [3, 4]. Similar to other herpesviruses, it establishes a life-long latency and persistence, and cannot be cleared by the immune system. The viral genome persists in a dormant form predominantly in the CD34^+^ hematopoietic progenitor cell population, which is resident in the bone marrow. Latent HCMV can be reactivated when the progenitor cells differentiate into macrophages or dendritic cells, and disseminate the virus to multiple cell types in different organs [5–7]. HCMV encoded proteins regulate adaptive immune responses to evade immune recognition and avoid elimination in its host through complex immunologic, metabolic and molecular interactions (Figures 1–2) [8]. While both primary infection and a reactivated HCMV infection rarely causes clinical symptoms in healthy individuals with a robust immune system, the virus may cause life-threatening disease in immunosuppressed patients. HCMV undergoes high mutation rates wherefore many viral genotypes exist. *In vitro* HCMV wild type strains rapidly lose some genes necessary for their persistence *in vivo,* which potentially affects their pathogenic potential. A comparison of the structure of both laboratory- and clinical HCMV strains is illustrated in Figure 3.

## HCMV IN HUMAN CANCERS

During the past years, a significant association between HCMV and human malignancies has been reported by several independent research groups. This has been a controversial field due to parallel negative studies failing to detect HCMV in tumor tissues when adjusted techniques for virus detection were not employed [9–11]. In addition, several groups have proposed that HCMV detection may vary with time due to increasing vulnerability of viral DNA for degradation during the sample storage [12]. Using optimized methods, many research groups demonstrate high prevalence of HCMV in breast, colon, and prostate cancer, rhabdomyosarcoma, hepatocellular cancer, salivary gland tumors, neuroblastoma and brain tumors (medulloblastoma and glioblastoma (GBM)) [13–19]. Over 90% of these tumors were positive for HCMV proteins and/or nucleic acids when exploiting methods such as *in situ* hybridization, PCR, electron microscopy, DNA and RNA sequencing, immunostaining of tissue specimens, flow cytometry analyses of tumor cells from surgical resections and western blot analysis [20]. Also, most neoplastic cells in sentinel lymph nodes of > 90% of breast cancer [19, 21], as well as 98% of brain metastases of colon and breast cancers contain HCMV proteins and/or nucleic acids [22]. The virus infection is restricted to tumor cells and some inflammatory cells and does not spread to adjacent normal cells.

## HCMV CAN PROMOTE ALL THE STEPS OF HALLMARKS OF CANCER

Hallmarks of cancer describe central, neoplastic processes, that are involved in tumor initiation and progression [23–27]. In addition to the earlier defined cellular oncogenic changes, the modern, wider concept of hallmarks of cancer brings in the complexity of tumor microenvironment and presence of cancer causing inflammation, as essential onco-modulatory mechanisms [27], which relates tumor initiation directly to infections by oncogenic viruses. HCMV encodes from 170 [28] to 750 proteins [29], and several of the HCMV encoded gene products are detected in tumors. Many of these gene products, especially the gene products expressed early during the HCMV life-cycle, regulate processes related to the hallmarks of cancer (as earlier reviewed by Herbein G. [30]). Expression of HCMV immediate early (IE) proteins (encoded by UL122 and UL123 genes), leads to a dysregulated cell cycle; and IE1 and IE2 gene products can promote immortalization by activating sustained hTERT telomerase activity and by blocking TNF-α mediated apoptosis [31, 32]. HCMV encoded early proteins also interfere with key cellular factors including retinoblastoma protein family (Rb), cyclins, p53, Wnt, PI3K/Akt, and NF-κB, and thereby they affect cell cycle control, cellular differentiation, proliferation, apoptosis and metabolism [33–35]. Another HCMV related gene, that is expressed during both latent and lytic HCMV infections, is US28, one of the four viral G protein-coupled receptors encoded by this virus. Constitutive US28 signaling results in activation of the hypoxia inducible factor-1α/pyruvate kinase M2 (HIF-1α/PKM2) axis, COX2 and 3-inducible nitric oxide synthase (STAT3-iNOS) mediating secretion of vascular endothelial growth factor (VEGF), and cellular motility [36–38]. US28 activates signaling pathways, that are involved in cell proliferation, survival, migration, angiogenesis and inflammation [36, 38–41]. Several studies have demonstrated mutagenic effects of HCMV encoded proteins IE1, pp65 and pp71 by inducing chromosomal aberrations, DNA breaks and disrupted DNA repair pathways [42–46].

**Figure 4:**
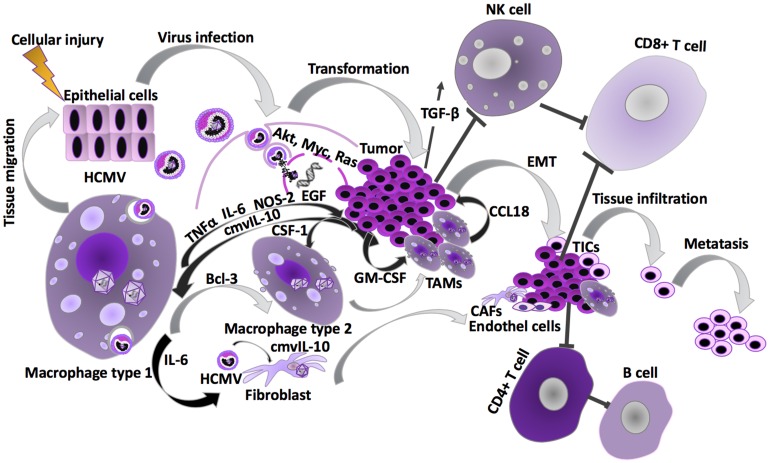
HCMV effects during carcinogenesis. Cancer predisposing risk factors are known to cause cellular injury, which in turn activates normal inflammatory response. HCMV can be reactivated as the latently infected monocytes differentiate into macrophages during migration as a part of this inflammatory response. The classically activated macrophages (M1) carrying a re-activated virus infection, can then infect other cell types, such as fibroblasts, endothelial and epithelial cells, which are more permissive to lytic HCMV infections. HCMV infected cells promote inflammatory and angiogenic secretome, that paracrinally, by intercellular signaling through secretion of cytokines, such as IL-6, TGFβ, GM-CSF and cmvIL-10, induce haemangiogenesis, lymphangiogenesis, cell proliferation as well as immune evasion/immunosupression. HCMV infection in the epithelial cells is evidenced to cause transformation to tumor cells [69]. The presence of HCMV infection in the tissue macrophages (M1) or secretion of GM-CSF by the tumor cells can result in activation of M2 macrophage differentiation pathway. Presence of M2 macrophages favor a pro-tumoral microenvironment due to their matrix-remodeling and anti-inflammatory properties [110, 111]. These immunosuppressive M2 macrophages display a phenotype that is closely related to tumor-associated macrophages (TAMs), of which presence in the tumors is known to play an important prognostic value. In addition, tumors contain a subpopulation of cancer cells, called cancer stem cells or tumor initiating cells (TICs), that have undergone an epithelial-mesenchymal transformation (EMT). The close vicinity of TAMs and cancer cells undergoing EMT at the invasive front of tumors, suggests that these cell types might interact mutually [5, 102]. The TAM-like macrophage phenotype secretes CCL5 stimulating EMT and migration of the cancer cells, and thereby increases the invasiveness and metastasis of the tumor [127]. Despite the transformation and tumor forming process involving epithelial cells, TAMs, TICs, endothelial cells and cancer associated fibroblasts (CAFs), the HCMV infection contributes to disarm the NK cells and adaptive immune responses (see Figure 2). NK cells activation of the cytotoxic T-cell responses displays a crucial function in the cell-mediated first-line host responses against viral infections and cancer initiation [128–130]. NK cells are also involved in antibody-dependent cellular cytotoxicity by B cell activation through CD4+ T cells [120].

HCMV-pp65, operates further to disable intrinsic cellular immune responses [47]. Another tegument protein known as pUL48, is an HCMV encoded deubiquitinase enzyme (HCMV-DUB) that inhibits synthesis of I-IFNs, an anti-cancer factor, by deubiquitinating several signaling molecules such as TNF receptor-associated factor (TRAF)-6 and -3, interleukin-1 receptor-associated kinase-1 (IRAK1), interferon regulatory factor (IRF)-7 or stimulator of interferon genes (STING), that play a key role in anti-viral innate immunity [48]. The viral proteins located in the HCMV US2-US11 region downregulate HLA class I- and class II-dependent antigen presentation to T cells [49, 50]. This will negatively influence activation of CD4+ T cell responses, activation of humoral immune response and CD8+ cytotoxic T cell elimination of the virus infected cells. In parallel, to counteract NK cell dependent cell lysis, the HCMV encoded HLA class I homolog UL18 can bind to the NK cell inhibitory receptor NKG2A/CD94 and expression of HLA-E, a non-classical HLA protein, is upregulated to inhibit NK cell activation [51–53]. HCMV further enhances production of immunosuppressive factors in the tumor microenvironment, such as transforming growth factor (TGF)-β and IL-10, and activation of regulatory T cells [54, 55]. TGF-β also directly inhibits NK cell effector functions. In addition, HCMV exhibits a powerful immunosuppressive effect by expressing an IL-10 homologue, cmvIL-10, which can promote maturation of pro-tumoral M2 macrophages and counteract the proper maturation of dendritic cells [56, 57] (Figure 4). Other lytic cycle immunomodulatory genes include: UL37/vMIA; pUL144, a Tumor Necrosis Factor receptor homolog; pUL128, a CC-like chemokine that modulates monocyte activity.

The previously recognized human onco-viruses are able to fulfill the first definitions of the hallmarks of cancer, such as essential alterations in the cell physiology, that are required for the cellular transformation. HCMV´s role in tumors has traditionally been depicted as oncomodulatory, i.e. with an ability to affect tumor cells to become more aggressive by enhancing cellular proliferation, survival, immunosuppression, angiogenesis, invasion and by creating a pro-inflammatory environment [58]. This latter role is indeed highly relevant, since functions of the tumor micro-environment have recently become recognized as key elements in tumor progression and metastasis in addition to the transforming ability of the virus.

## HCMV CAN TRANSFORM EPITHELIAL CELLS

While involved in all steps of the hallmarks of cancer as discussed above, several HCMV proteins can potentially participate to initiate cellular transformation. Some historical evidence, published between 1976–1993, has reported that HCMV is able to induce *in vitro* transformation of human embryonic lung fibroblasts and rodent fibroblasts [59–64]. Early evidence suggested that fragments of the viral genome can transform cells [65]; expression of HCMV IE or US28 proteins have been shown to lead to cellular transformation under certain conditions. Expression of US28 led to tumor development in mouse 3T3 cells. Targeted US28 expression in the colon of transgenic mice resulted in tumor transformation, especially under inflammatory challenge [66]. Expression of the HCMV IE proteins together with the adenovirus E1A protein promoted cellular transformation through a “hit and run” mechanism [67], as IE was no longer detected in the transformed cells.

Recent studies by Lepiller *et al*. showed that infection of primary hepatocytes and HepG2 cells with a newly isolated strain, HCMV-DB, led to tumor colony formation in soft agar cultures [68]. HCMV-DB, was also able to transform human primary mammary epithelial cells (HMECs), which led to tumor development in immunodeficient mice [69]. These recent results confirm data from the old studies, demonstrating that perhaps only particular HCMV clinical strains exhibited oncogenic properties and led to tumor development in immunodeficient mice [62]. A common feature of these virus strains seems to be their lack of a rapid lytic infection, which is expected by most HCMV strains, and would protect cells from oncogenic transformation. A non-lytic infection will allow for many virus mediated mechanisms to act in an oncomodulatory and oncogenic fashion without killing the cells, and allow for oncogenic transformation.

In primary HMECs infected with the clinical isolate HCMV-DB, the molecular requirements for oncogenic immortalization and transformation *in vitro* were met: inactivation of p53 and Rb, telomere maintenance, acquisition of constitutive mitogenic signals provided by Ras/cMyc, Akt activation, STAT3 activation and cyclin D1 overexpression with a consequence of enhanced cellular proliferation [69]. The transformed HMECs displayed an HCMV RNA signature and gave rise to rapidly growing triple negative breast tumors when injected in NOD scid gamma (NSG) mice. A similar HCMV RNA signature was detected in tumor biopsies of patients with breast cancer, but not in healthy breast tissue [69]. Our team has recently shown that HCMV is highly prevalent in triple negative breast tumors, and a newly published clinical study demonstrate a potential role of HCMV infection in the development of a triple negative breast cancer phenotype [70]. Taken together, these results suggest that certain HCMV strains may not only have an oncomodulatory capacity, but in some cellular context exhibit direct oncogenic and tumor promoting mechanisms.

## NON-LYTIC OR LATENT HCMV INFECTIONS MAY LEAD TO ONCOGENIC TRANSFORMATION

Establishment of HCMV latency is associated with post-translational modifications of histones that regulate repression of major immediate-early promoter (MIEP) activity in a cell differentiation-dependent manner. The complex interaction between cellular transcription factors and a highly chromatinized histones around the MIEP promotes inhibition of IE gene expression and preserves HCMV latency [71–73]. Latency is established in myeloid lineage progenitor cells and secure life-long persistence of the infection [5, 74]. Several viral gene products are expressed, and latently infected cells control the virus survival through a number of immunosuppressive mechanisms [75]. In contrast to the latent stage when the histones around the MIEP sequence are heavily methylated and prevent expression of the lytic genes, the MIEP sequence was no longer detectable in transformed HMECs infected with the HCMV-DB wild-type strain after 14 days in the soft agar cultures [69]. Such scenario would have profound effects on the virus life cycle. Most early and late genes are controlled by the IE proteins that are transcription factors [13, 14, 22, 70, 76]. If viral IE and early genes are expressed without viral replication and in the absence of cell lysis, they may contribute to cellular proliferation and survival of infected tumor cells and lead to oncogenic transformation [69]. This may be mediated by infections of HCMV strains causing a non-lytic infection. Most HCMV strains are not able to replicate efficiently in transformed cells such as hepatocellular carcinoma HepG2 cells or in fibroblasts expressing SV40 T antigen (TAg) and oncogenic H-Ras [68, 77]. This was proposed to be caused by a block of viral cellular entry and nuclear delivery of viral DNA and pp65 protein through TAg mediated downregulation of the HCMV receptor platelet-derived growth factor receptor (PDGFR), which impacted on accumulation of the major immediate early (MIE) transcripts. Overexpression of PDGFR and IE transcripts increased the HCMV gene expression, but did not rescue the production of infectious virions [77]. The reason for this phenomenon is unknown, but may be dependent on the requirement of the virus to halt cells in G_0_ phase of the cell cycle to proceed into a lytic phase [78]. Genomic sequence analysis of HCMV strains in glioblastoma suggests that the virus is not replicating in these cells either, as much fewer mutations than expected during HCMV replications were observed [79]. Thus, the virus may express many viral genes while lacking some critical factor for virus DNA replication and/or assembly to produce new viral progeny. Nevertheless, the viral proteins expressed could act in oncomodulatory and oncogenic processes.

## HCMV INFECTS SEVERAL CELL TYPES IN THE TUMOR

HCMV infection of freshly isolated glioblastoma multiforme (GBM) cells *in vitro* induced stemness, and maintained cells in an undifferentiated stem-like state [80]. In addition, the HCMV infection is shown to be present in both microglia and in CD133 expressing cells and promotes a M2 macrophage phenotype differentiation in tumors [57]. This scenario may increase the aggressiveness of GBM tumors, and enhanced number of CD133 stem cells in tumors is indeed associated with poor prognosis [80]. Consistently, recent data from Krenzlin *et al.* shows that mouse CMV (MCMV) promotes glioblastoma growth *in vivo*. Reactivation was shown in perivascular, intra-tumoral pericytes, that seem to be protected from elimination by tumor induced immunosuppression [81]. MCMV infection was also present in the tumor cells as shown by immunostaining [81]. This multicellular HCMV infection might explain the confusion regarding the expression of HCMV latent/lytic proteins in tumors. The poorly differentiated, stem-cell-like cancer cells may contain latent HCMV infection and only express a set of viral proteins, whereas the infection in some other surrounding cell-types is productive, and produce late HCMV proteins as was shown by Bahador *et al*. [12]. The immunosuppression induced by the HCMV infected tumor cells through secretion of GM-CSF [82], and other immunosuppressive mediators such as TGF-β and cmvIL-10 [54, 55], creates a tumor micro-environment that protects the productively infected cells from destruction by disarming NK-cells and impairing the CD8+ cytotoxic T cell mediated tumor elimination (Figure 4) [56, 57]. The productive infection in the tumor microenvironment would further promote a stem-cell-like state of tumor cells [82] and enhance tumor aggressiveness. Thus, the multicellular HCMV infection creates a vicious circle, resulting in a non-lytic infection followed by transformation of the epithelial cells, and HCMV reactivation in M1 macrophages promoting an M2/TAM shift in surrounding macrophages, which could drive the neoplastic process. Supporting these assumptions, glioblastoma patients exhibit signs of immunosuppression that are similar to those observed in HCMV infected individuals, (Figure 2) [83], and enhanced viral load in glioblastoma tumors is associated with poor patient outcome [84].

## TREATMENT OF ONCOGENIC HCMV INFECTIONS

Novel approaches for treatment of HCMV infections are areas of great research interest. No vaccines aimed at ameliorating the severity of disease and preventing HCMV infections have so far been successful in achieving durable and protective immunity [85, 86]. Several new promising vaccine strategies against HCMV have been developed and evaluated *ex vivo* and in animal models [82, 87, 88]. A pp65 mRNA dendritic cell vaccination approach was recently used in glioblastoma patients and indicated highly improved survival rates in a small subset of patients [89, 90]. Likewise, adoptive immunotherapy with HCMV specific T cells show promising results with improved outcome in individual GBM patients [91]. These observations suggest that immune activating strategies against HCMV may be of benefit for HCMV positive glioblastoma patients.

The accessibility of antiviral therapy such as ganciclovir, valganciclovir, cidofovir and foscarnet against HCMV has offered a great improvement in the treatment and prevention of HCMV infection and has ensued considerably better prognosis for immunocompromised patients. Consecutively, the clinical benefit of most of these agents is restricted by low oral bioavailability, related toxicities, and the risk of developing drug resistance with prolonged use. Most antiviral agents target actively replicating viruses, but not their latent states. However, latently expressed protein products of EBV, and potentially also HCMV, may have significant effects on cancer biology. Induction of lytic gene expression results in elimination of virus-infected cells, and increases their vulnerability to antivirals and most likely also to immunotherapy. Chemotherapeutic drugs, such as doxorubicin, gemcitabine, cisplatin, etoposide, 5-fluorouracil, and paclitaxel have shown their potential to induce EBV reactivation [86, 92]. Combination therapy may therefore be a favorable approach in the treatment of herpes virus associated malignancies by induction of viral lytic gene expression followed by exposure of the tumor cells to antiviral drugs and immunotherapy.

Some earlier promising data arising from the studies in glioblastoma patients indicates, that the effect of antiviral treatments of HCMV positive cancers should be investigated further. Animals with HCMV positive human medulloblastoma or neuroblastoma xenograft tumors, that were treated with anti-HCMV drugs, showed significantly reduced tumor sizes, when treated with anti-HCM drugs than placebo treated animals [81, 93, 94]. Our interdisciplinary team has considerable experience in treating cancer patients with valganciclovir, the mainstay treatment for clinical HCMV infections. We first performed a pilot double blinded study on 42 glioblastoma patients. This study was underpowered and failed its primary end point. However, survival benefits were noted and additional patients were prescribed valganciclovir as add on to standard therapy for glioblastomas. In 2013, we reported our first follow up data; treatment of 50 glioblastoma patients who received anti-CMV treatment as an add-on to standard adjuvant therapy at Karolinska University Hospital, Sweden, demonstrated a 2-year survival of 70% among 40 patients receiving 6 months of anti-viral therapy, and as high as 90% survival among patients with continuous treatment (*n* = 25) compared with 18% in contemporary controls (*n* = 137). Strikingly, the latter treatment group showed a median OS of 56.4 months compared with 13.5 months in controls (*P* < 0.0001) [19]. Overall survival at four years was 27.3% in anti-HCMV treated patients versus 5.9% in controls [95]. Today, we have treated 135 glioblastoma patients with valganciclovir, and we continue to observe highly improved survival rates among glioblastoma patients with both primary and recurrent disease. We are currently preparing for initiation of a clinical trial to assess the effect of valganciclovir treatment as add on to standard therapy in glioblastoma patients in a double blinded multicenter trial. First patients are expected to be enrolled in June 2019.

Most likely patients would benefit from combined therapies targeting HCMV. Both COX-1 and COX-2 inhibitors are efficient anti-viral drugs that prevent HCMV replication [96, 97]. A synergistic treatment effect together with antiviral therapy was observed with a COX-2 inhibitor in medulloblastoma, which resulted in a 72%-97% reduced medulloblastoma tumor growth in an animal model and *in vitro* [13]. Cidofovir reduced glioblastoma growth and improved glioblastoma survival in recently published MCMV glioblastoma mice model [81]. New anti-CMV therapies are under development. Letermovir is a new type of antiviral treatment, that can offer a treatment choice for patients with resistance to classical antivirals, and was approved for clinical use against HCMV in US in 2017 [98]. Additionally, a very recent study reported the repurpose of manidipine dihydrochloride (MND), a calcium antagonist, which is clinically approved to treat hypertension, as a new anti-HCMV agent [99]. It remains to be seen if combination of different set of antiviral-strategies against HCMV can give better efficacy and less resistance development without compromising safety and treatment efficacy aspects. In HIV patients, the virus was not controlled with one anti-viral drug, but needed combined antiviral therapy for clinical control of virus replication [100].

## SHALL HCMV BE CONSIDERED AS AN ONCOGENIC VIRUS?

The precise role of HCMV as a cancer-causing agent in human cancers has not been clarified, and the virus is not included in the list of oncogenic viruses [9, 69]. However, in addition to being present in tumors, the biological properties of HCMV may fulfill the criteria for an oncogenic virus, when established modified standards based on Hill’s 9 criteria, proposed by Fredericks and Relman [101], are used. The existing literature reports, that i) HCMV can be found in over >90% of human epithelial tumors such as breast, colon, ovarian and prostate cancer, rhabdomyosarcoma, hepatocellular cancer, salivary gland tumors, neuroblastoma and brain tumors (medulloblastoma and glioblastoma) [13–19, 21, 102–104]. ii) HCMV infected cells are confined within tumors and metastasis, and not found in adjacent normal tissues [19, 22, 105]. iii) The level of HCMV infection in the tumors correlates negatively with the positive disease outcome [22, 70, 84, 106]. iv) Treatment of the infection with antiviral therapy in HCMV positive cancer patients indicate improved prognosis and potentially represents a new effective anti-cancer strategy [13, 19]. v) HCMV gene products regulate multiple tumorigenic cellular pathways and processes related to all the “Hallmarks of cancer” [27, 101, 107]. Several HCMV encoded proteins exhibit biological properties that are directly related to cellular transformation and tumor development [33–35, 69, 80]. vi) HCMV infection shows a broad cellular tropism, and is present in tumor epithelial cells, macrophages, endothelial cells and sometimes in the stroma cells of the tumors [70, 108–112]. HCMV differs from other onco-viruses on this point, but as discussed earlier in this paper, the broad tissue tropism of HCMV may have strong oncogenic impact on both tumor cells, through consequences in the tumor microenvironment and on the immune system. vii) The reproducibility of the results (i-vi) have historically been variable, and arguments against a role of HCMV in cancer have disputed that several studies have failed to confirm the association of HCMV with glioma, breast cancer, or other malignancies by polymerase chain reaction (PCR), *in situ* hybridization, and immunohistochemical methods [9–11].

Increasing recent evidence suggests a need to reconsider why some of the previous results have been inconsistent, and whether they reflect some common characteristics of the HCMV. It is now well known that antigen retrieval protocols are needed to unmask HCMV proteins if tumors are embedded in paraffin, while viral proteins are readily detected in frozen sections. *In situ* hybridization easily detects viral DNA, whereas PCR and sequencing methods generally fail to consistently detect the HCMV genome in tumor specimens. In addition, as already mentioned, the infection is different in permissive cells, in which the virus replicates and produce infectious virus, compared to in transformed cells *in vivo* or *in vitro*. HCMV infected tumor cells often express cytoplasmic IE proteins, which are rarely observed in other infected tissues and not in *in vitro* studies. These observations suggest an alternative behavior of HCMV in tumor cells or the existence of a tumor associated unique virus strains. Rescuing of the virus from tumor cells of primary tumors, primary tumor cell cultures or established tumor cell lines [113] seems to be difficult or impossible. In contrast, HCMV is easily retrieved from infected tissue specimens during an acute infection. The oncogenic related HCMV infection in the epithelial cells might therefore resemble to that of latent HCMV infection, since the true biological character of latency is simply, that these are cells not making infectious virus while still expressing numerous viral genes [75]. This latent gene expression might become uncontrolled due to the changes on the MIEP sequence: It is also possible that HCMV transformed tumor cells lose the entire, or parts of the viral genome, or develop numerous mutations during the transformation process, and that tumors arise through a “hit and run mechanism” [69]. This scenario would similarly explain the difficulty in finding viral DNA in tumor cells by PCR [69], while the viral genome is detectable with large probes used *in situ* hybridization. Macrophages or stroma cells in close vicinity to the tumor may still contain an intact HCMV genome, and represent the small amounts of viral DNA reported in tumors by several investigators [13, 81, 113–116]. However, after years of work trying to resolve this issue, we instead favor the hypothesis that unresolved technical problems account for the lack of detection of HCMV DNA in tumors by PCR and sequencing methods. This may be due to high level of genetic variance of CMV and/or difficulties of Taq polymerases to read the viral DNA or RNA (cDNA) code [81].

Another point of criticism against a potential role of HCMV in tumorigenesis is that, in contrast to all other known onco-virus, most HCMV strains are unable to transform normal human cells *in vitro*. However, there might be several, natural explanations for some failed efforts to show transforming properties of the HCMV in the past. It is well established that HCMV changes its characteristics quickly under *in vitro* conditions and behaves differently compared to *in vivo* situations [117]. The fact that different virus infected cells may cooperate during a transformation process *in vivo,* may explain failures to induce oncogenic transformation in *in vitro* experiments, as it has been difficult to recapitulate the *in* *vivo* requirements for this process in experimental models. Target cells may also need to acquire accumulation of relevant somatic mutations before an HCMV infection could lead to oncogenic transformation. Equally, mutations may lead to a non-lytic infection and enhanced oncogenic capacity. Most importantly, alternative strains of HCMV have been poorly characterized for oncogenic capacity, although they may be of significant importance *in vivo*. So far, wild-type strain HCMV-DB indicate a possible ability to transform human epithelial cells *in vitro* [69], likewise a clinical strain used by Fred Rapp´s group in the 1970th exhibited similar properties [62]. What characteristics these strains may have, making them potentially oncogenic and perhaps only in certain cellular context, is unknown. Questions therefore arise whether certain HCMV strains are tumor causing, and if so, what the carrier prevalence of such altered strains is in the human population? Such viruses are likely not leading to lytic infection in cell types in which oncogenic transformation may occur. They may also be highly cell associated, or defective, and thereby difficult to culture under conditions generally used for culturing HCMV *in vitro*. In support of this hypothesis, an HCMV strain from a paraganglioma was rescued with protocols not generally used for virus propagation [118]. As infections with specific cancer associated HCMV strains could explain why not all HCMV infected individuals develop cancer, studies to clarify the identity of HCMV strains in cancer specimens are promptly needed.

In conclusion, a profound understanding of the link between HCMV infections, cancer initiation and/or cancer progression as well as clarification of the identity of the cancer associated viruses might help to identify individuals with higher cancer risk and to identify patients who could benefit from anti-viral strategies. To realize this goal, clinical trials are urgently needed to explore the efficacy and safety of antivirals in combination with classical cancer treatments such as surgery, radiation therapy, chemotherapy and immunotherapy in patients with HCMV associated malignancies, to assess whether HCMV provides a target for precision-based oncology treatment protocols.
